# A Species-Wide Inventory of NLR Genes and Alleles in *Arabidopsis thaliana*

**DOI:** 10.1016/j.cell.2019.07.038

**Published:** 2019-08-22

**Authors:** Anna-Lena Van de Weyer, Freddy Monteiro, Oliver J. Furzer, Marc T. Nishimura, Volkan Cevik, Kamil Witek, Jonathan D.G. Jones, Jeffery L. Dangl, Detlef Weigel, Felix Bemm

**Affiliations:** 1 Department of Molecular Biology, Max Planck Institute for Developmental Biology, 72076 Tübingen, Germany; 2 Howard Hughes Medical Institute, Chevy Chase, MD 20815, USA; 3 Department of Biology, University of North Carolina, Chapel Hill, NC 27599-3280, USA; 4 Center for Research in Agricultural Genomics (CRAG), CSIC-IRTA-UAB-UB, 08193 Barcelona, Spain; 5 Department of Biology, Colorado State University, Fort Collins, CO 80523, USA; 6 The Sainsbury Laboratory, University of East Anglia, Norwich Research Park, Norwich NR4 7UH, UK; 7 Milner Centre for Evolution & Department of Biology and Biochemistry, University of Bath, Bath BA2 7AY, UK

**Keywords:** NLR, innate immunity, plant immunity, disease resistance genes, SMRT sequencing, RenSeq, sequence capture, targeted enrichment, genomics, integrated domains

## Abstract

Infectious disease is both a major force of selection in nature and a prime cause of yield loss in agriculture. In plants, disease resistance is often conferred by nucleotide-binding leucine-rich repeat (NLR) proteins, intracellular immune receptors that recognize pathogen proteins and their effects on the host. Consistent with extensive balancing and positive selection, NLRs are encoded by one of the most variable gene families in plants, but the true extent of intraspecific NLR diversity has been unclear. Here, we define a nearly complete species-wide pan-NLRome in *Arabidopsis thaliana* based on sequence enrichment and long-read sequencing. The pan-NLRome largely saturates with approximately 40 well-chosen wild strains, with half of the pan-NLRome being present in most accessions. We chart NLR architectural diversity, identify new architectures, and quantify selective forces that act on specific NLRs and NLR domains. Our study provides a blueprint for defining pan-NLRomes.

## Highlights

• Species-wide NLR diversity is high but not unlimited
• A large fraction of NLR diversity is recovered with 40–50 accessions
• Presence/absence variation in NLRs is widespread, resulting in a mosaic population
• A high diversity of NLR-integrated domains favor known virulence targets

## Graphical Abstract

**Figure:**
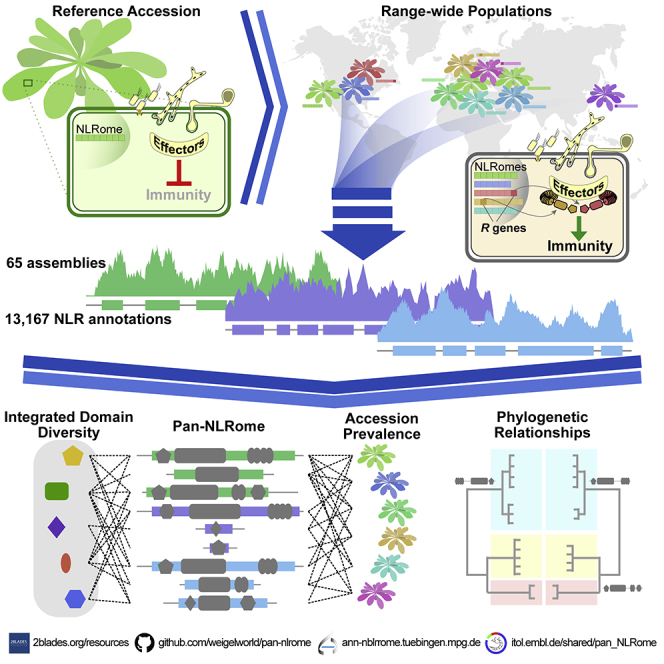

## Teaser

In plants, NLR proteins are important intracellular receptors with roles in innate immunity and disease resistance. This work provides a panoramic view of this diverse and complicated gene family in the model species *A. thaliana* and provides a foundation for the identification and functional study of disease-resistance genes in agronomically important species with complex genomes.

## Introduction

Plant immunity relies critically on a repertoire of immunity receptors whose diversity has been shaped by eons of plant-microbe coevolution. Two classes of receptors can activate immune signaling: cell-surface proteins that recognize microbe-associated molecular patterns (MAMPs) and intracellular proteins that detect pathogen effectors (Dangl et al., 2013). A large portion of the latter class comprises nucleotide-binding leucine-rich repeat receptors (NLRs). These are encoded by highly polymorphic genes that represent the majority of genetically defined disease-resistance loci (Jones et al., 2016, Kourelis and van der Hoorn, 2018, Monteiro and Nishimura, 2018), with hundreds of NLR genes being found in the typical flowering plant genome (Shao et al., 2016). Most plant NLRs contain a central nucleotide-binding domain shared by Apaf-1, resistance proteins, and CED4 (NB-ARC, hereafter NB for simplicity), and either a Toll/interleukin-1 receptor (TIR) or coiled-coil (CC) domain at the N terminus (Jones et al., 2016, Monteiro and Nishimura, 2018). Proteins with similar arrangements of functional domains are involved in host defenses of animals and fungi (Jones et al., 2016, Uehling et al., 2017). Similar to animal NLRs, plant NLRs appear to form inflammasome-like structures, or resistosomes, that control cell death following pathogen recognition (Wang et al., 2019a, Wang et al., 2019b).

Pathogen recognition by plant NLRs generally involves one of at least three main mechanisms (Kourelis and van der Hoorn, 2018). NLRs can detect pathogen effectors indirectly by monitoring how they modify host targets, known as NLR guardees. Alternatively, direct detection of pathogen effectors occurs either through interaction of effectors with any of the three canonical NLR domains or through interaction of effectors with integrated domains (IDs) that resemble bona fide host targets and act as target decoys.

Because of their importance in ecology and breeding, there has been much interest in defining inventories of NLR genes at different taxonomic levels. These efforts have revealed that the number of NLR genes across species varies from fewer than a hundred to over a thousand (Yue et al., 2012, Zhang et al., 2016a), they have supported phylogenetic reconstruction of key NLR lineages (Shao et al., 2016), and they have greatly expanded the universe of ID-containing NLRs (Bailey et al., 2018, Gao et al., 2018, Kroj et al., 2016, Maqbool et al., 2015, Sarris et al., 2016, Shao et al., 2016), which are excellent candidates for engineering new pathogen resistances (Helm et al., 2019, Kim et al., 2016, Kourelis et al., 2016, Monteiro and Nishimura, 2018).

While there has been substantial progress at higher taxonomic levels, a thorough understanding of NLR diversity within species has unfortunately been hindered by the extraordinarily polymorphic nature of the gene family, and its extensive pervasive presence-absence polymorphic variation even between closely related individuals. Early intraspecific diversity studies revealed patterns of allelic and structural variation consistent with adaptive evolution and balancing selection for subsets of NLR genes (Bakker et al., 2008), fitting a model of co-evolution of host and pathogens. Some loci can have many different haplotypes, in some instances even reflecting true allelic series (Allen et al., 2004, Dodds et al., 2006, Rose et al., 2004). A further complication is that many NLR genes are arranged in clusters with extensive copy-number variation (Chae et al., 2014, Cook et al., 2012, Leister et al., 1998, Meyers et al., 1998, Noël et al., 1999). Together with ubiquitous presence-absence polymorphisms, this implies that reference genomes likely include only a fraction of distinct NLR genes within a species, which in turn has made it impossible to obtain a clear picture of NLR diversity based on resequencing efforts. To remedy this gap in our knowledge of NLR gene evolution, resistance gene enrichment sequencing (RenSeq) has been developed, which is especially powerful when combined with long read technology (Witek et al., 2016a).

Here, we present a substantial step toward defining the full NLR repertoire, or pan-NLRome, and its variability in the reference species *Arabidopsis thaliana*, by analyzing a highly curated diversity panel of 64 accessions. Despite the extreme diversity of NLR complements when comparing only a few individuals, discovery of the pan-NLRome of this species approached saturation with about 40 well-chosen accessions. The sequences we obtained allow us to define the core NLR complement, chart integrated domain diversity, describe new domain architectures, assess presence-absence polymorphisms in non-core NLRs, and map uncharacterized NLRs onto the *A. thaliana* Col-0 reference genome. Together, our work provides a foundation for the identification and functional study of disease-resistance genes in agronomically important species with more complex genomes.

## Results

### The Samples

**Figure 1:**
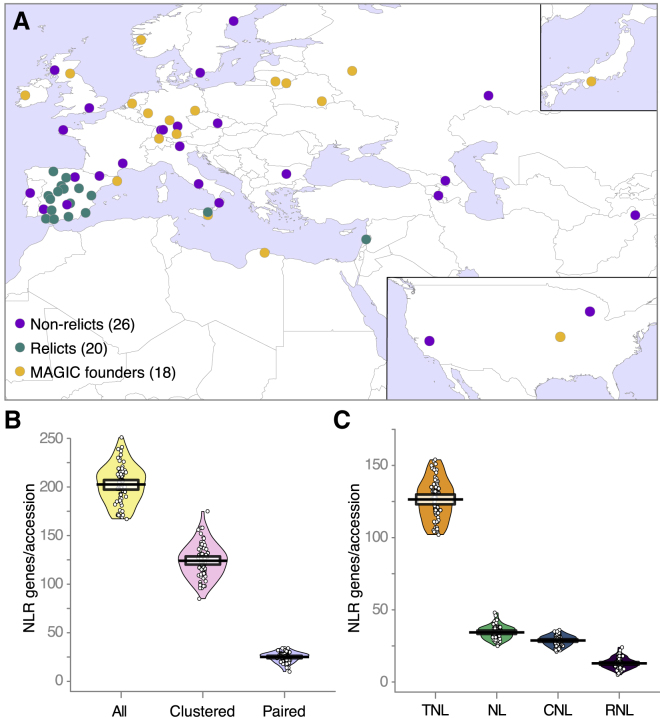
Overview of NLR Complements in 64 Accessions (A) Accession provenance. 1001 Genomes relicts, non-relicts, and MAGIC founders. (B) Total number (yellow) as well as number of clustered (rose) and paired (purple) NLRs in each accession. Solid black lines, means; transparent horizontal bands, Bayesian 95% highest density intervals (HDIs); circles, individual data; full densities shown as bean plots. (C) Number of NLRs in different structural classes in accessions. Orange, TNLs; green, NLs; blue, CNLs; purple, RNLs (purple). Related to Figure S1 and Table S1.

We selected 64 *A. thaliana* accessions for RenSeq analysis. Of those, 46 were from the 1001 Genomes Project collection (1001 Genomes Consortium, 2016), with 26 representing non-relict accessions, several of which have informative disease-resistance phenotypes, and 20 belonging to relict populations characterized by an unusually high amount of genetic diversity. A further 18 of the accessions are founders of multiparent advanced generation inter-cross (MAGIC) lines (Kover et al., 2009) (Figure 1A; Table S1). One MAGIC accession was sampled twice due to a mislabeled seed stock (total number of datasets was thus 65).

### NLR Discovery

RenSeq baits were designed to hybridize with 736 NLR-coding genes from multiple Brassicaceae, including *A. thaliana*, *A. lyrata*, *Brassica rapa*, *Aethionema arabicum*, and *Eutrema parvulum*. RenSeq was combined with single-molecule real-time (SMRT) sequencing to reconstruct 65 NLR complements, resulting in 13,167 annotated NLR genes, with a range of 167 to 251 genes per accession (Figure 1B). We report the annotated RenSeq sequences and identifiers also for the reference accession Col-0, but for downstream analyses, we used TAIR10 and Araport 11 identifiers and sequences for Col-0 (Cheng et al., 2017, Lamesch et al., 2012).

**Figure S1:**
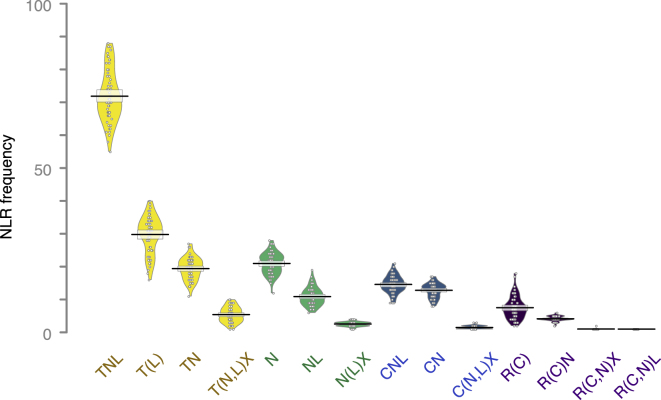
NLR Frequency for Different Subclasses, Related to Figure 1 NLRs are grouped by domain content: T (TIR), N (NB), C (CC), R (CC_R_), and X (all IDs). Domains in parentheses are not present in all members of that group. Domain order is not considered. Mean is shown as a solid black horizontal line and the 95% Highest Density Intervals (HDI; points in the interval have a higher probability than points outside) are shown as transparent bands around the sample mean. Individual data points plotted as open circles and full densities shown as bean plots.

Adopting a definition of NLR clusters as genes within 200 kb of each other in the genome (Holub, 2001), 47%–71% of NLR genes in each accession were located in such clusters. A particularly interesting subset of NLR genes are those in head-to-head orientation, termed paired NLRs (Narusaka et al., 2009, Saucet et al., 2015). We found 10–34 such NLRs per accession. NLRs were grouped into the four classes: TIR-NLR (TNL), CC-NLR (CNL), CC_R_-NLR (RNL), and NB-and-LRR-only proteins (NL), based on canonical NLR domains TIR, CC, RPW8-like coiled-coil (CC_R_), NB, and leucine-rich repeats (LRRs). Most NLR genes in each accession were TNLs, which also were the most variable in overall gene number, followed by NLs, CNLs, and RNLs (Figures 1C and S1).

### Diversity of NLR Domain Architectures

**Figure 2:**
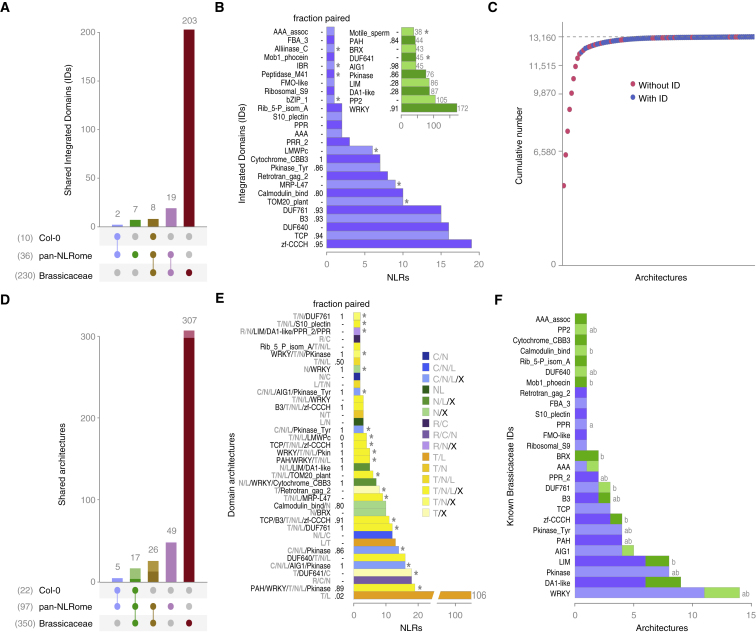
Diversity of IDs and Domain Architectures (A) UpSet intersection of IDs in the Col-0 reference accession, pan-NLRome, and 19 other Brassicaceae. (B) ID distribution, with IDs not reported before from *A. thaliana* in blue and previously known IDs in green. Asterisks indicate IDs not reported before from other Brassicaceae. (C) Cumulative contribution to the pan-NLRome by different domain architectures, ranked from largest to smallest. (D) UpSet intersection of architectures shared between Col-0 reference accession, pan-NLRome, and 19 other Brassicaceae. Darker colors indicate architectures with IDs. (E) 38 new *A. thaliana* architectures not found in the Col-0 reference and represented by more than one gene. Asterisks indicate architectures also not found in 19 other Brassicaceae. (F) Newly described (blue) and previously known (green) architectures containing the 27 overlapping Brassicaceae IDs (see A). “a*”* and “b” indicate IDs as defined in (Kroj et al., 2016) and (Sarris et al., 2016), respectively. Related to Figure S2 and Table S2.

Of the 13,167 NLR genes, 663 encoded at least one non-canonical NLR domain, or ID, representing 36 distinct Pfam domains (Figures 2A–2C; Tables S2A and S3A). Individual accessions had 5–17 IDs distributed across 4–16 NLR genes, including several IDs not reported before in *A. thaliana* or other Brassicaceae. Of the 36 IDs, 29 were already known from other Brassicaceae including the *A. thaliana* reference accession Col-0 (Figures 2A and 2B; Tables S2A–S2C and S3B). Nine had been reported concordantly in two major genome-wide NLR-ID surveys (Kroj et al., 2016, Sarris et al., 2016), namely WRKY, phloem protein 2 (PP2), protein kinase, paired amphipathic helix repeat (PAH), unknown domain DUF640, B3, protein tyrosine kinase, PPR repeat family 2, and alliinase, of which five occur in genetically linked paired NLRs (Figure 2B; Table S2A). Rediscovery of these nine IDs is of particular relevance, since they are enriched for domains similar to known effector targets (Kroj et al., 2016, Mukhtar et al., 2011, Sarris et al., 2016, Weßling et al., 2014). Note that singleton IDs, defined as those that were found in only one NLR gene model in one accession, were not considered further, to minimize the effects of potential annotation artifacts on our analyses.

**Figure S2:**
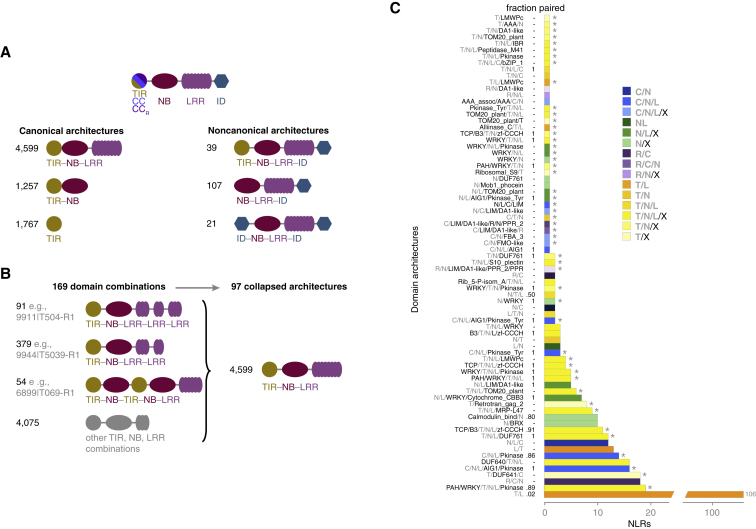
Schematic Representation of NLR Domain Architecture Diversity and Simplification of Consecutively Repeated Domains, Related to Figure 2 (A) Examples of NLR domain architecture diversity. (B) Reduction of TNL domain combinations by collapsing duplicated/repetitive domains. Analogous strategies were applied to CNL, RNL and NL classes. (C) Full set of NLR architectures not described before for *A. thaliana*, including architectures found in only one gene. Asterisks indicate 49 architectures not reported from other Brassicaceae, or in the reference accession Col-0.

A hallmark of NLRome diversity across species is the variation in the relative fraction of different domain architectures (Li et al., 2015, Sarris et al., 2016, Shao et al., 2016, Yue et al., 2012, Zhang et al., 2016a). We identified 97 distinct architectures in the *A. thaliana* pan-NLRome, of which only 22 were found in the Col-0 reference genome and only 48 had been reported in Col-0 or other Brassicaceae (Figures 2D and S2; Table S2D). Fewer than a third of architectures, 27, corresponded to different configurations of the canonical TIR, CC, CC_R_, NB, and LRR domains, even though they accounted for the vast majority, 95%, of NLRs. The remaining 5% of NLRs had all at least 1 of 36 different IDs, with most of the ID-containing architectures not seen before in *A. thaliana* (Figures 2B, 2C, and S2C). Half of the new *A. thaliana* architectures, 38 out of 75, were represented by more than one gene (Figure S2C). Many of these, 17, comprised paired NLRs with at least one ID (Figure 2E). All but 1 of the 175 NLR genes with new architectures contained an ID, and together they made up 1.3% of the pan-NLRome (Figures 2D and 2E; Tables S2D–S2F). Finally, 12 IDs were found in more than one new architecture (Figure 2F; Tables S2E and S2F), reflecting the recycling of a limited set of IDs into new domain arrangements. Coincidentally, since it is likely that IDs are derived from proteins repeatedly targeted by pathogen effectors, their identification provides leads for the identification of new pathogen effector targets, even though only the TCP domain has been found in a large interaction screen with diverse pathogen effectors (Weßling et al., 2014).

### The Pan-NLRome

**Figure 3:**
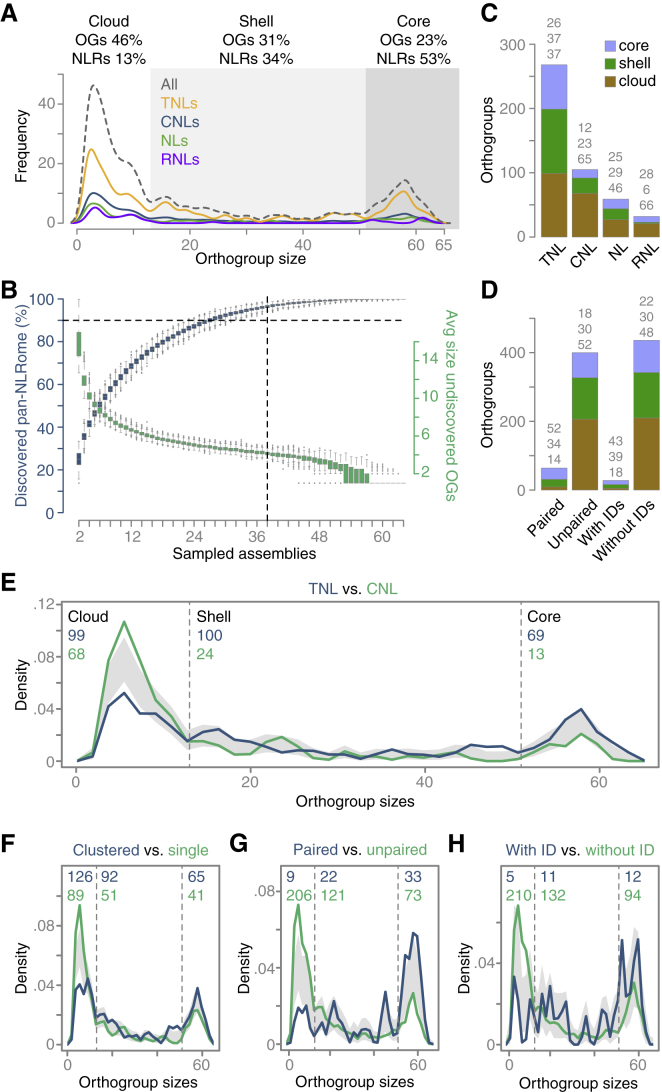
Orthogroup Sizes, Saturation, and Distribution of Core, Shell, and Cloud NLRs (A) OG size distribution (without singleton OGs). (B) Saturation of pan-NLRome discovery. Blue indicates fractions of pan-NLRome that can be recovered from randomly drawn sets of accessions of different sizes (with 1,000× bootstrapping). Horizontal dashed line indicates 90% of pan-NLRome discovered. Green indicates average sizes of OG that remain undiscovered with accession sets of different sizes. Vertical dashed line indicates that 95% of the pan-NLRome can be recovered with 38 accessions (1,000 bootstraps). (C) OG-type-specific distribution of NLR classes in cloud (brown), shell (green), and the core pan-NLRome (blue). Percentages for each on top. (D) OG-type-specific distribution of paired and unpaired NLRs and NLRs with and without IDs in cloud (brown), shell (green), and core (blue). Percentages on top. (E–H) Comparison of OG size density distributions across different contrasting NLR subsets. The blue and green numbers denote the total number of OGs in the cloud, shell, and core for each of the four contrasting subsets shown. Gray bands indicate the ranges in which the OG size density distributions would not be significantly different from each other, determined with a bootstrap approach.

To understand both the variation in NLR content and diversity of NLR alleles, we clustered NLRs from different accessions into orthogroups (OGs) based on sequence similarity. Only a little more than 10% of all NLRs, 1,663, were singletons, with the rest, 11,497, falling into 1 of 464 OGs. The OG size distribution of these 464 non-singleton OGs is shown in Figure 3A. Of the OGs, 95% could be discovered with 38 randomly chosen accessions (Figure 3B). Additional sampling only recovered OGs with three or fewer members, indicating that the pan-NLRome we describe is largely saturated.

OGs were classified according to size, domain architecture, and structural features. The core NLRome could be defined with merely 106 OGs (23%), comprising 6,080 (53%) genes, that were found in at least 52 accessions. A slightly higher number, 143 (31%) OGs, with 3,932 (34%) genes, were found in at least 13 but fewer than 52 accessions, a class that we considered the shell NLRome. Finally, 46% of all OGs, 215, which included 1,485 (13%) genes, were found in 12 or fewer accessions, constituting the cloud NLRome (Figure 3A).

The majority of OGs, 58%, were TNLs, in agreement with TNLs being the prevalent NLR class in the Brassicaceae (Guo et al., 2011, Meyers et al., 2003, Peele et al., 2014, Zhang et al., 2016b), 22% were CNLs, 7% were RNLs, and 13% were NLs (Figure 3C). Specific TNLs were missing from accessions on average less often than CNLs, reflected in CNL OGs being much more likely to be part of the cloud pan-NLRome (Figures 3C and 3E). 64 OGs included genetically paired NLRs, and 28 contained members with an ID, with almost all belonging to the shell or core NLRome (Figures 3D, 3G, and 3H). In general, NLR clusters and pairs as well as ID-containing NLRs were not only widely distributed in the population, but both were also on average more conserved than unpaired NLRs or those without IDs (Figures 3D–3H).

We tested for each shell or core OG whether the topology of its phylogeny could be linked to available metadata, such as subclass membership or expression across accessions. With stringent filtering criteria, we found 56 such associations for 68 OGs, with 16 OGs having multiple associations (Table S4I). The most frequently associated metadata types were relic classification (50; e.g., OG40.1), domain architecture (11; e.g., OG172.1), NLR subclass (10; e.g., OG174.1), and the pattern of surrounding transposable elements (10; e.g., OG160.1). An association with resistance to *Albugo candida* (*Ac*Ex1) was found twice (OG16.10 and OG284.1). None of the OGs showed a strong association with expression pattern, population classification, or geographic origin of accessions.

Previous studies, such as the one of Kuang and colleagues (Kuang et al., 2004), found that some NLR loci feature signs of frequent sequence exchange between paralogs (type I), whereas others have a more obvious allelic pattern (type II). We could identify clear allelic series for 86% of OGs, but 14% of OGs appeared initially as overclustered (i.e., including non-allelic genes). Such overclustering could reflect high sequence similarity because of frequent exchange between paralogs or recent duplication events, or in other words, being consistent with potential type I loci. Among these initially overclustered loci, we found the *RPP8/RCY1/HRT*, *RPP4/5*, and *RPP7/TuNI* OGs. These loci constitute all three known examples in *A. thaliana*, where different paralogs confer resistance to different classes of pathogens, or distinct effectors from the same pathogen, a sign of neo-functionalization (Asai et al., 2019, van der Biezen et al., 2002, Liu et al., 2015, Takahashi et al., 2002). This suggests that type I-like loci are important for the evolution of new pathogen-recognition specificities.

### Genomic Placement of Non-reference OGs

**Figure S3:**
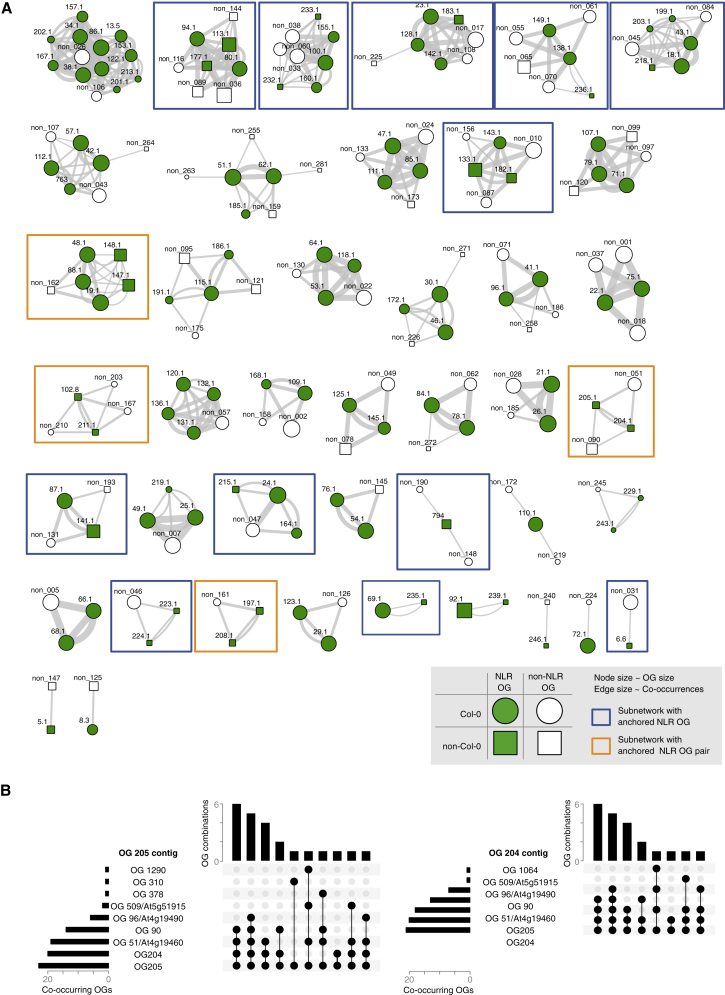
OG Combinations, Related to Figure 4 (A) Co-occurrences network for NLR (no prefix) and non-NLR (prefix “non_”) OGs on the same contigs in **≥** 10 accessions. Similar networks were found for higher or lower thresholds. Blue boxes highlight NLR OGs without a Col-0 allele, orange boxes highlight paired OGs without a Col-0 allele. (B) Co-occurrence of the paired, head-to-head NLRs OG205 (TCP-B3-TIR-NB-LRR-Zf) and OG204 (TIR-NB-LRR), which are not found in Col-0 or in Ler. Grey, non-NLR OGs.

**Figure 4:**
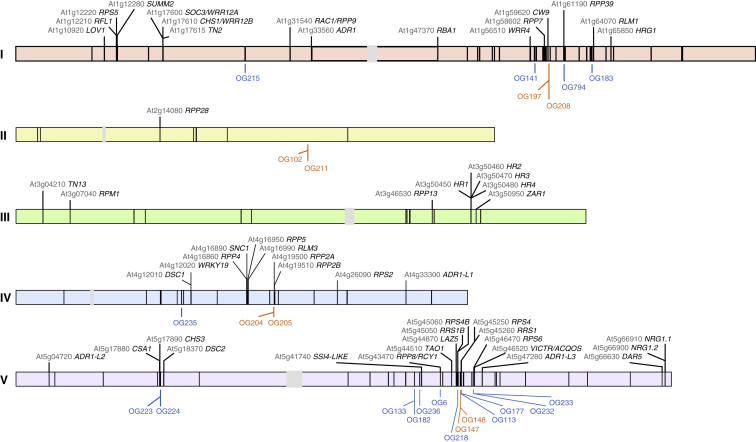
Genomic Location of *NLR* Genes in the Reference Assembly The five *A. thaliana* chromosomes are shown as horizontal bars with centromeres in gray, and reference NLRs are shown as black line segments. Text labels are shown only for functionally defined Col-0 NLRs. Anchored OGs found in at least 10 accessions are shown below each chromosome. Orange, paired OGs; blue, other anchored OGs. Related to Figure S3 and Table S3C.

296 OGs were missing from the Col-0 reference genome, with 6 belonging to the core, 205 to the cloud, and 85 to the shell pan-NLRome. To anchor these OGs to the reference genome, we looked for co-occurrence of such OGs on the same contig as NLR or non-NLR OGs with a Col-0 reference allele (Table S3C). With a threshold of 10 accessions, we derived 42 co-occurrence subnetworks (Figure S3), which allowed us to anchor 24 of 132 non-reference OGs present in at least 10 accessions. Non-reference OGs were mostly linked to regions known from Col-0 to contain NLRs (Table S3C), which was expected, since our baits for enrichment were based on NLR sequences. However, OG102 and OG211 were found in a region not known before to contain NLRs (Figures 4 and S3). Newly anchored OGs included one CNL and three TNL pairs, one of which was the ID-containing sensor-type OG205, which was found in head-to-head orientation with the executor-type OG204 (Figure S3).

### Pan-NLRome Diversity

**Figure S4:**
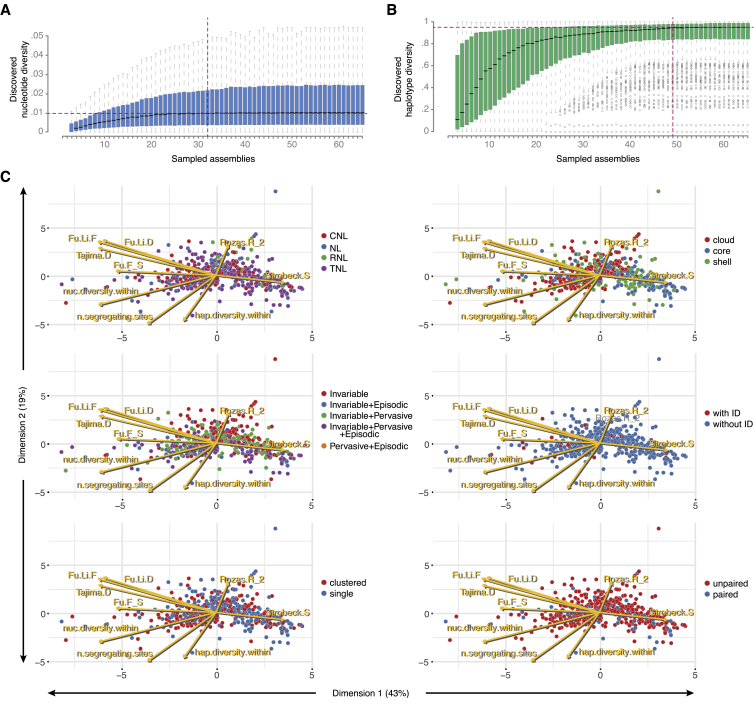
Saturation of Diversity Discovery and PCAs of Population Genetics Statistics, Related to Figure 5 (A and B) Fraction of nucleotide and haplotype diversity that can be recovered from a randomly drawn set of accessions with different set sizes (with 1000x bootstrapping). Horizontal dashed lines indicate 90% of diversity found. Vertical dashed line indicates number of accessions with which 95% of diversity can be recovered (1,000 bootstraps). (C) Principal component analysis carried out on 10 population genetics statistics, nucleotide diversity (pi), haplotype diversity, Fu and Li’s D, Fu and Li’s F, Tajima’s D, Rozas’ R_2_, Strobeck’s S and number of segregating sites.

**Figure 5:**
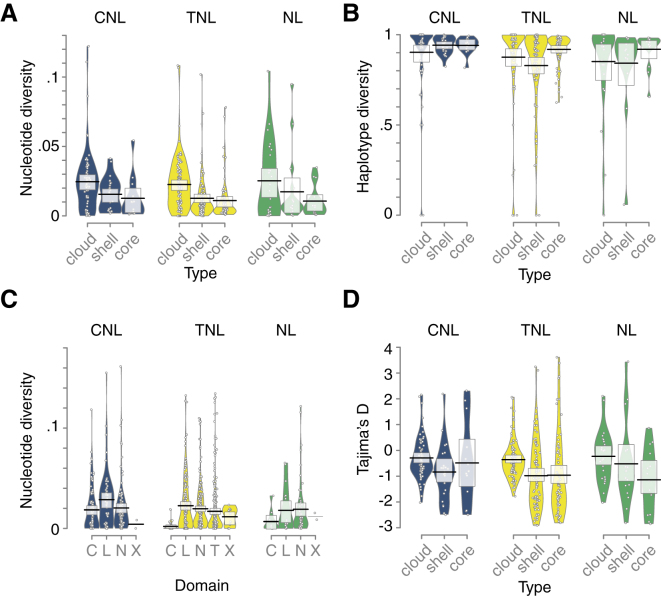
Diversity and Selection across the Pan-NLRome RNL OGs are not shown because of the low number of OGs in this class. (A) Nucleotide diversity (average pairwise nucleotide differences) by OG type and NLR class. (B) Haplotype diversity (average pairwise haplotype differences) by OG type and NLR class. Large values indicate a high chance of finding two different haplotypes when two randomly chosen members of a given OG are compared. (C) Nucleotide diversity distribution in different domain types. The NL class included a few OGs where a minority of members had an identifiable CC domain; hence the CC class and the NL class overlapped. (D) Tajima’s D, a measure of genetic selection, by OG type and NLR class. Related to Figure S4.

In an orthogonal approach to classifying NLR genes according to their architectures, we assessed sequence diversity as an indication of the evolutionary forces shaping the pan-NLRome. Average nucleotide diversity reached 95% saturation already with 32 randomly selected accessions. In contrast, haplotype diversity saturated only with 49 accessions, reflecting that new haplotypes emerge not only by mutation but also by intragenic recombination and gene conversion, for which we found evidence in three-quarters (74%) of OGs (Table S4G). This is in agreement with long-standing observations that intragenic recombination can contribute to functional diversification of NLR genes (McDowell et al., 1998) (Figures S4A and S4B). Compared to non-clustered OGs, clustered OGs had significantly higher nucleotide diversity (Figure S4), consistent with relaxed selection after gene duplication in these clusters (Ohno, 1970). Even though the different NLR classes had very different profiles when it came to presence-absence polymorphisms (Figure 3B), average nucleotide diversity within OGs was similar for CNLs, TNLs, and NLs (Figure 5A). It was lowest in RNLs, consistent with their function as conserved helper NLRs (Monteiro and Nishimura, 2018), but because this was the smallest group, this difference was not statistically significant. Haplotype diversity was also similar for different NLR classes, being highest in core OGs (Figure 5B). This is consistent with OGs that were present in many accessions having haplotypes with similar population frequencies and any random pair of accessions therefore often representing different haplotypes. Nucleotide diversity decreased from cloud to core OGs (Figure 5A), consistent with within-haplotype nucleotide diversity for common haplotypes being comparatively low. A few cloud OGs and a couple of shell TNL OGs stood out because of their ultra-low haplotype diversity, indicative of OGs that, when present, constitute only a single haplotype, without any geographic bias in the distribution of accessions with these OGs. Such haplotypes could be maintained by a conserved but rarely encountered selective pressure.

When considering different NLR protein domains, the highest diversity was found in LRRs across all major classes and subclasses, consistent with LRRs being more likely than other domains to be involved in ligand binding and to be under diversifying selection (Goritschnig et al., 2016, Krasileva et al., 2010, Shen et al., 2003) (Figure 5C). Combining population genetics statistics for a principal-component analysis (PCA) revealed that more than 60% of the variance could be explained by the first two principal components (Figures S4C–S4H). However, none of the known properties, such as OG size, OG prevalence, selection type, NLR class, or the presence of IDs or a potential partner, explained the first two principal components (Figures S4D–S4H), suggesting a complex interplay of different factors driving NLR evolution. Tajima’s D values, which can indicate balancing and purifying selection (Tajima, 1989), were similarly distributed across different NLR classes, with all classes containing extremes in both directions (Figure 5D), although negative Tajima’s D values, indicative of an excess of rare alleles, were most common in TNLs.

**Figure 6:**
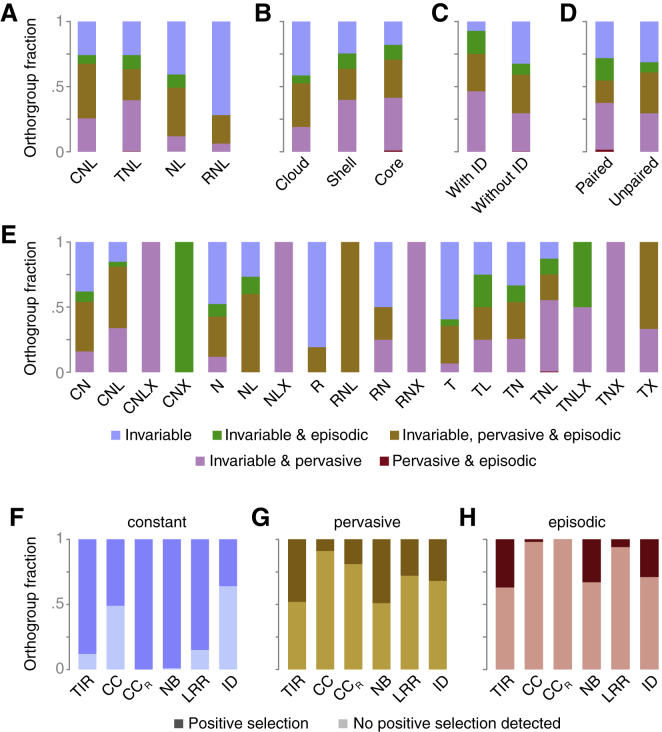
Selection Landscape of the Pan-NLRome (A–E) Fraction of different positive selection categories grouped by NLR class (A), OG type (B), ID status (C), paired NLR status (D), or NLR subclass (E). An OG was considered if at least one positive selected site of a given class was detectable. (F–H) Fractions of OGs inferred to be under constant (F), pervasive (G), or episodic (H) selection or without positive selection detected, grouped by annotated protein domains. Related to Figure S5.

**Figure S5:**
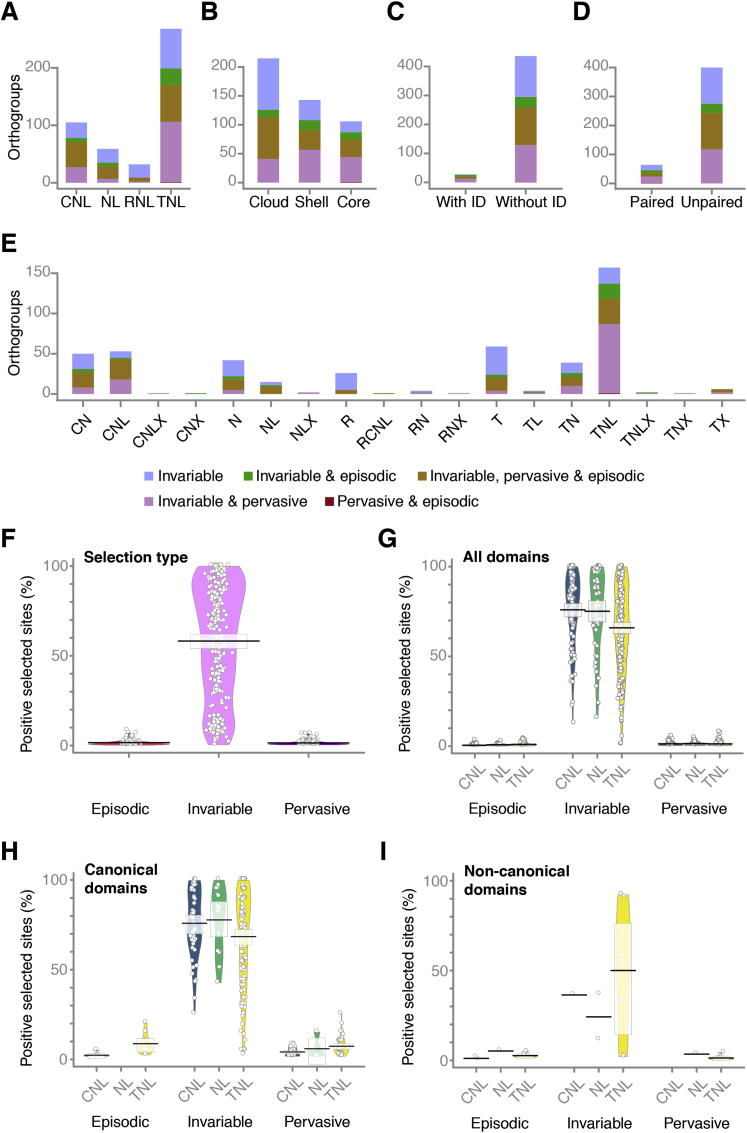
Positive Selection Landscape of the Pan-NLRome, Related to Figure 6 (A–E) Number of OGs in different selection classes grouped by NLR class (A), OG type (B), ID status (C), paired NLR status (D), or NLR subclass (E). An OG was considered if at least one positive selected site of a given class was detectable. (F) NLR coverage with different types of positively selected sites. (G–I) Domain coverage with positively selected sites.

A selection analysis on individual branches identified 131 OGs with at least one branch under episodic positive selection (Table S4H). Most OGs belonged to the core (50) or shell (73) NLRomes. A subset of 32 OGs included branches with members that had different metadata associations. Most overlaps were found for associations with patterns of surrounding transposable elements (10; OG159.1) or NLR subclasses (9; OG106.1), followed by expression patterns (7; OG115.1), population (7; OG70.1) and relic classifications (5; OG77.1), or the geographic origin (4; OG21.1). A single OG included a selected branch linked to resistance to *A. candida* (*Ac*Ex1; OG173.1). Site-specific selection analyses revealed 543 core and shell OGs that had likely experienced constant (46%), pervasive (30%), or episodic (24%) positive selection (Figures 6A, 6B, and S5). Invariable codons, indicating constant purifying selection, could be found across all types (e.g., core, shell), classes (e.g., TNLs, CNLs), and pair-status (e.g., paired, unpaired) (Figures 6A–6D). Subclasses showed an uneven pattern of positive selection (Figure 6E), and sites under constant positive selection were more likely in TIR, CC_R_, NB, and LRR than in CC and ID domains (Figure 6F). Pervasive and episodic positive-selection patterns appeared predominantly in NB and TIR domains (Figures 6G and 6H). A few OGs stood out because of the large fraction of codons of annotated protein domains under positive selection, including *RPP13*, which is well known because of its allelic series that confers race-specific downy mildew resistance (Bittner-Eddy et al., 2000, Rose et al., 2004) (Figure S5). Sites under positive selection were also found in 11 IDs, including WRKY, TCP, B3, and DA1-like domains (Figure 6C). Notably, invariant sites were detected in the WRKY domains of all three OGs containing a WRKY and in a surprisingly high proportion of sites in the BRX domains of the RLM3-containing OG (Table S4A). We conclude that positive selection is widespread in the core NLRome, being most prevalent in canonical NLR domains.

### Linking Diversity to Known Function

**Figure 7:**
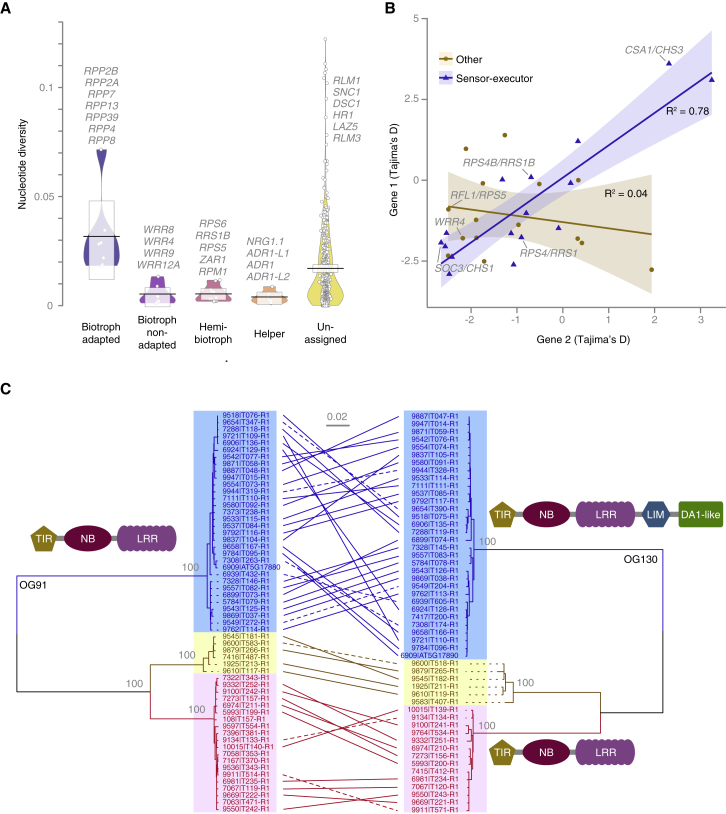
Effects of Pathogen Lifestyle on Diversity and NLR Pairing on Selection and Co-evolution (A) Effect of pathogen lifestyle on nucleotide diversity for characterized resistance genes. Gray floating text indicates examples for each category. (B) Correlation of Tajima’s D values in sensor-executor and other pairs. (C) Maximum-likelihood phylogenetic trees of two OGs 91 and 130, which form a sensor-executor pair (Xu et al., 2015). Bootstrap support (100 iterations) indicated at major nodes. OG130 includes a clade with LIM and DA1-like IDs and a clade without. Scale bar indicates substitutions per site. Genes from the same accession are connected by lines, with solid lines indicating presence on the same assembly contig. Related to Figure S6.

Because NLRs that had been experimentally implicated in resistance to biotrophic pathogens showed enhanced diversity, we sorted OGs by resistance to adapted biotrophs (*Hyaloperonospora arabidopsidis*), non-adapted biotrophs (*Brassica*-infecting races of *A. candida*) (Cevik et al., 2019) and hemibiotrophs (mostly *Pseudomonas* spp.). OGs that provide resistance against adapted biotrophs were significantly more diverse than other categories (Figure 7A; ANOVA and Tukey’s HSD p < 0.01), suggesting that host-adapted biotrophic pathogens are driving diversification of NLRs more than other pathogens. That RNL helper NLRs had low diversity is consistent with their requirement to function with several sensor NLRs (Bonardi et al., 2011, Castel et al., 2019, Wu et al., 2017).

**Figure S6:**
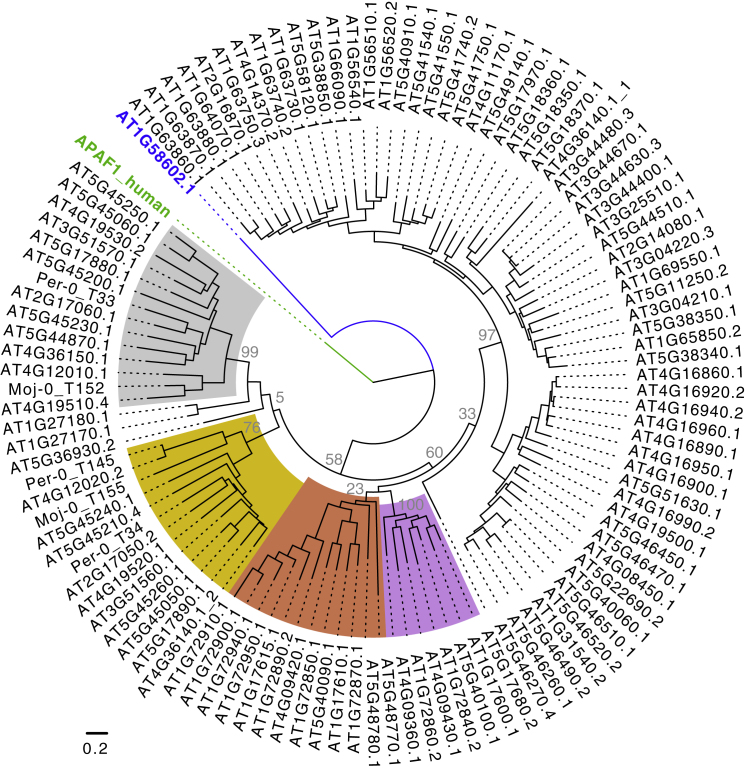
Phylogenetic Tree of NB Domain Alignments of TNLs to Delineate Sensor-Executor Pairs, Related to Figure 7 The paired NLRs RPS4-like (executors, silver) and RRS1-like (sensors, gold) as well as SOC3-like (executors, light purple) and CHS1-like (sensors, brick) defined distinct subclades of TNLs. The NJ phylogeny was built from manually refined MUSCLE alignments of NB domains (∼240 amino acids) of Col-0 proteins plus selected additional representatives of OGs inferred to be paired, but absent from the Col-0 reference. NB domains from human APAF1 (green) and the *A. thaliana* CNL AT1G58602 (blue) were included as outgroups. The WAG maximum likelihood method allowing for 3 discrete Gamma categories was used. AT4G36140 contains two distinct NB domains, both of which were included; the second NB domain of AT4G36140 groups with other RRS1-like NB domains. Support from 100 bootstraps shown at major nodes. Scale bar indicates substitutions per site.

Among the OGs with the lowest Tajima’s D values, a prominent example was *RPM1*, which confers resistance to a hemibiotrophic bacterial pathogens, and for which an ancient, stably balanced presence-absence polymorphism across *A. thaliana* is well established (Stahl et al., 1999). OGs that provide resistance to adapted biotrophs tended to have higher Tajima’s D values, indicating that they experience not only diversifying but also balancing selection. Tajima’s D values within sensor-executor pairs encoded in head-to-head orientation were correlated, whereas other closely linked NLR genes or random pairs were not (Figures 7B and S6; Table S4B). As an example, two OGs with high Tajima’s D values were the paired NLRs *CSA1* (OG91) and *CHS3* (OG130). *CHS3* featured two very different groups of alleles distinguished by the presence of LIM and DA1-like IDs (Xu et al., 2015). This allelic pattern was perfectly mirrored by the one for *CSA1*, the paired executor partner NLR of *CHS3*, even though it lacks IDs—evidence for the importance of within-pair specificity (Figure 7C).

## Discussion

We have defined a nearly complete species-wide repertoire of the gene family that encodes NLR immune receptors in the model plant *A. thaliana*. Our first important observation was that the pan-NLRome inventory became >98% saturated with any 40 of the 64 accessions analyzed. It was known before that there was excessive variation at some NLR loci, such that in the small number of accessions in which the relevant genomic region was analyzed in detail, every accession was very different, including significant presence-absence variation (Noël et al., 1999, Rose et al., 2004). That our pan-NLRome saturated with a relatively small set of accessions indicates that the number of divergent loci is not unlimited. Our success in pan-NLRome discovery almost certainly reflects also the choice of our accession panel based on extensive prior knowledge of diversity within the species (1001 Genomes Consortium, 2016). Our study thus not only provides guidance for future efforts in other species in which genome-wide diversity has been characterized, but it is also good news for the informed safeguarding of germplasm of crop species and their wild relatives. Nevertheless, pan-NLRome discovery efforts in crops will also depend on the type of pathogen that is the most significant threat for a particular crop, since we observed the highest sequence diversity in NLR genes that provide resistance to evolutionarily adapted biotrophic pathogens.

Another important observation is that the diversity of IDs is substantially greater than what one might have guessed based on the Col-0 reference genome alone. IDs are thought to allow hosts to rapidly accrue the ability to recognize pathogen effector proteins. Most ID-containing NLRs that have been functionally characterized are found in paired orientation. In these pairs, the ID member functions as pathogen sensor, and the other member as signaling executor (Cesari et al., 2014, Le Roux et al., 2015, Narusaka et al., 2009, Sarris et al., 2015, Saucet et al., 2015, Xu et al., 2015, Zhang et al., 2017), with both members contributing to repression and activation of NLR signaling (Ma et al., 2018). Considered the primary interface for pathogen effector interaction, we anticipated that sensor NLRs would exhibit a stronger signal of selection than their executor partners. In contrast to this expectation, the correlation between Tajima’s D values of such paired NLRs supports a co-evolutionary scenario whereby mutations in the sensor component lead to compensatory changes in the executor, or vice versa. On the codon level, however, many IDs did exhibit signals of positive selection. The allelic data that we present, particularly in pairs where there is polymorphism or presence-absence in the ID, present an opportunity for further experimental studies of NLR pair-complex dynamics.

Half of the 22 most commonly found IDs did not occur in an arrangement indicative of sensor-executor pairs. An open question is whether these function with unlinked executor partners or whether they can function as dual sensor-executor proteins. Within the *A. thaliana* pan-NLRome, we identified three key groups of IDs, derived from defense-related TCP, WRKY, and CBP60 transcription factors, all of which are represented as IDs in sensors of the class defined by the NLR RRS1. The TCP domain is particularly interesting, as TCP transcription factors are targeted by effectors from divergently evolved pathogens (Mukhtar et al., 2011, Sugio et al., 2014, Weßling et al., 2014, Yang et al., 2017). IDs in effector targets have the potential to provide new avenues for engineering of NLR specificity, for example through TCP swaps or inclusion of known effector-interacting platforms from TCP14 (Yang et al., 2017), in analogy with protease cleavage site swaps (Helm et al., 2019, Kim et al., 2016).

In aggregate, our work not only provides a panoramic view of NLR genes in a species, but it also provides the first step for more detailed investigations of NLR diversity within populations at different scales. In particular, it enables rapid assessment of NLR variants in local populations within the geographic range covered by accessions used here. In addition, our pan-NLRome provides a baseline for the study of geographic regions that have only recently been recognized as harboring additional genetic diversity, such as Africa, Macronesia, and Central Asia (Durvasula et al., 2017, Fulgione and Hancock, 2018, Fulgione et al., 2017, Zou et al., 2017). And despite the apparent saturation of NLR diversity at the level of OG diversity, we not only expect that the universe of very rare NLR genes is likely substantial, but also that more subtle variation, primarily at the allelic level, can be discovered with the analysis of additional accessions. Similarly, natural *A. thaliana* pathogen incidence and diversity will almost certainly help to interpret the OG variation we report here (Durvasula et al., 2017).

Finally, the pan-NLRome of the selfing diploid species *A. thaliana* will serve as a basis for comparison with the pan-NLRome of obligate out-crossers in the genus such as *A. lyrata*, as well as autopolyploids such as many *A. arenosa* individuals, allopolyploids such as *A. suecica*, and domesticated paleopolyploid *Brassica* species (Clauss and Koch, 2006, Hollister et al., 2012, Novikova et al., 2016). For example, in polyploids, NLR gene deletions might be more frequent because of fitness tradeoffs (Grant et al., 1998). As another example, out-crossers have generally greater diversity than selfers (Wright et al., 2013), but it is unknown whether this also applies to NLR genes, as increased diversity at these loci might increase the risk of costly intra-immune system conflict (Chae et al., 2016, Karasov et al., 2017). Understanding what promotes and limits NLR diversity in different plants is an important prerequisite both for learning how wild species adapt to their biotic environment and for discovering how breeding can make crops more resilient to old and emerging pathogens (Durvasula et al., 2017).

## STAR★Methods

### Key Resources Table

**Table:** 

REAGENT or RESOURCE	SOURCE	IDENTIFIER
**Deposited Data**		

Raw data and assembled sequences	This paper	ENA:PRJEB23122
Genome browser	This paper	http://ann-nblrrome.tuebingen.mpg.de
GitHub pan-NLRome repository	This paper	https://github.com/weigelworld/pan-nlrome/
iTOL OG phylogenetic trees	This paper	https://itol.embl.de/shared/pan_NLRome

**Experimental Models: Organisms/Strains**		

*Arabidopsis thaliana*	(1001 Genomes Consortium, 2016)	Accession details and sequencing facilities: Table S1

**Oligonucleotides**		

Bait library: 20,000 synthetic 120 nt biotinylated RNA probes listed in Table S5B	This paper	Custom design from Mycroarray (Now Arbor Biosciences, MI, USA), Table S5B
Dual 8 bp barcoded adapters	This paper	Table S5A

**Software and Algorithms**		

CCS	(Travers et al., 2010)	https://github.com/PacificBiosciences/ccs
Canu	(Koren et al., 2017)	RRID:SCR_015880
MAKER	(Campbell et al., 2014)	RRID:SCR_005309
AUGUSTUS	(Stanke et al., 2006)	RRID:SCR_008417
SNAP	(Korf, 2004)	https://github.com/KorfLab/SNAP
RepeatMasker	Smit AFA, Hubley R & Green P. RepeatMasker Open-4.0. 2013-2015	RRID:SCR_012954
InterProScan	(Zdobnov and Apweiler, 2001)	RRID:SCR_005829
Coils	(Lupas et al., 1991)	RRID:SCR_008440
Paircoil2	(McDonnell et al., 2006)	https://cb.csail.mit.edu/cb/paircoil2/
DIAMOND	(Buchfink et al., 2015)	RRID:SCR_016071
orthAgogue	(Ekseth et al., 2014)	RRID:SCR_011979
mcl	(Enright et al., 2002)	https://micans.org/mcl/
T-Coffee	(Notredame et al., 2000)	RRID:SCR_011818
PAL2NAL	(Suyama et al., 2006)	http://www.bork.embl.de/pal2nal/
NLR-parser	(Steuernagel et al., 2015)	https://github.com/steuernb/NLR-Parser/
OD-seq	(Jehl et al., 2015)	https://github.com/hawk31/odseq/
FastME	(Lefort et al., 2015)	www.atgc-montpellier.fr/fastme/
R and RStudio, including packages: PopGenome, yarrr, viridis, factoextra, corrplot, magrittr, dplyr, plyr, fitdistrplus, lattice, gridExtra, grid, FactoMineR, cowplot, gridGraphics, ggplot2, easyGgplot2, ggpubr, RColorBrewer, reshape2, gsubfn, rworldmap, UpSetR, PerformanceAnalytics, karyoploteR, viridis and sm	(RStudio Team, 2015, R Development Core Team, 2008, Pfeifer et al., 2014, Conway et al., 2017)	RRID:SCR_001905
3seq	(Lam et al., 2018)	http://mol.ax/software/3seq/
aBSREL	(Smith et al., 2015)	https://www.datamonkey.org/absrel
ExaBayes version 1.4.1	(Aberer et al., 2014)	https://cme.h-its.org/exelixis/web/software/exabayes/

**Other**		

*A. thaliana* Col-0 transcripts and proteins from Araport database	(Cheng et al., 2017)	https://www.araport.org/downloads/
*Capsella rubella* and *Arabidopsis lyrata* gene models from Phytozome database	(Slotte et al., 2013, Hu et al., 2011)	https://phytozome.jgi.doe.gov/
*A. thaliana* expression data AtGenExpress (deprecated)	(Schmid et al., 2005, Kemmerling et al., 2011)	https://www.arabidopsis.org/portals/expression/microarray/ATGenExpress.jsp/

### Lead Contact and Materials Availability

Further information and requests for resources and reagents should be directed to and will be fulfilled by the Lead Contact, Detlef Weigel (weigel@weigelworld.org).

### Experimental Model and Subject Details

#### Accession selection

64 *Arabidopsis thaliana* accessions were used for this study, germplasm sources are listed in Table S1. Among the 1001 Genomes Project accessions, a subset known as ‘relicts’ is distinguished by its high genetic diversity (1001 Genomes Consortium, 2016). To maximize NLRome diversity, 20 relicts were included. To maximize phenotypic diversity, a set of 18 MAGIC founders were included (Kover et al., 2009, Scarcelli et al., 2007). The remaining accessions were chosen to maximize high diversity at the whole genome level, representing different genetic groups (1001 Genomes Consortium, 2016) (Table S1). The reference accession Col-0 was included for quality control purposes.

#### Accession verification

Routine seed stock genotyping prevents sample contamination (Pisupati et al., 2017). At a late stage of this project, 46 accessions were re-sequenced as part of a routine seed stock verification effort and the accessions were determined using SNPmatch (Pisupati et al., 2017). Three mis-labeled accessions were found in our dataset (1001 Genomes Project IDs 7063, 9911 and 9658). Their accession names and IDs were corrected (Table S1). For the sake of contiguity, their project IDs were not changed. 7063 was identified as 7186, which means there is one accession occurring twice in our analyses. The total number of datasets (referred to as accessions in the main text for simplicity) is thus 65.

### Method Details

#### SMRT RenSeq

Genomic DNA libraries were enriched for NLR sequences and sequenced using PacBio long read technology (Witek et al., 2016b). Library preparations were performed collaboratively in three labs (UNC, MPI, and TSL) with minor handling differences. DNA was extracted using either the DNeasy Plant Maxi kit (UNC) (QIAGEN, CA, USA), a custom high molecular weight DNA extraction protocol (MPI), or by grinding in Shorty buffer (20% 1M Tris HCl pH 9, 20% 2M LiCl, 5% 0.5M EDTA, 10% SDS, 45% dH2O), followed by phenol chloroform extraction and precipitation with isopropanol (TSL). Extracted DNA was fragmented to 2-5 kb using either Covaris red miniTubes (Intensity = 1, DutyCycle = 20%, Cycles per Burst = 1000, Treatment time = 600 s, Temperature = 20°C, Water level = 15, Sample volume = 200 μl) (TSL), or Covaris g-tubes using manufacturer’s instructions for a targeted size of 6 kb (UNC, MPI) (Covaris, MA, USA). DNA was purified using 0.4x AMPure XP beads (Beckman Coulter, IN, USA) according to manufacturer’s instructions.

Libraries were constructed using ‘NEBNext Ultra DNA Library Prep Kit for Illumina’ (New England Biolabs Inc, MA, USA). Sixteen accessions from TSL were prepared for multiplexed sequencing, by introducing custom barcoded adapters (dual 8 bp index) instead of the standard ones (Table S5A). For PCR amplification, 5-10 μl adaptor-ligated DNA was used together with 25 μl 2x KAPA HiFi HotStart ReadyMix, 1 μl Index and Universal PCR Primer, and 13-18 μl water (to a total volume of 50 μl) (Kapa Biosystems, MA, USA). Initial denaturation (94°C for 4 min) was followed by at least 8 cycles (denaturation: 94°C for 30 s, annealing: 65°C for 30 s, extension: 68°C for 4 min) and a final extension (68°C for 10 min).

For enrichment of NLR genes, 20,000 synthetic 120 nt biotinylated RNA probes (*bait library*), complementary to 736 known NLR genes from the reference genomes of *Arabidopsis thaliana* (Swarbreck et al., 2008), *Arabidopsis lyrata* (Hu et al., 2011), *Brassica rapa* (Wang et al., 2011), *Aethionema arabicum* (Haudry et al., 2013) and *Eutrema parvulum* (Yang et al., 2013) were designed (MYbaits; MYcroarray, MI, USA) (Table S5B). Where known, additional *A. thaliana* alleles were included, along with non-repetitive intron regions to improve capture of genes with introns > 350 bp. 100-500 ng of library DNA was hybridized with the baits using half of the reaction volume suggested in MYbaits v3.0 protocol, with the following modifications: For each reaction, hybridization mix was prepared using 10 μl Hyb#1, 4 μl Hyb#3, 0.4 μl Hyb#2 and 0.4 μl Hyb#4; library mix with 2.5 μl SeqCAP (Roche), 0.3 μl Block#3 and 3 μl gDNA library; capture mix with 2.5 μl bait library and 0.5 μl RNase block (MYcroarray, MI, USA). Following the manufacturer’s recommended cycling conditions, mixes were brought to a hybridization temperature of 65°C and 5 μl of the library mix and 5.5 μl of the hybridization mix were combined with the capture mix. After 16 to 24 hours hybridization, the enriched libraries were recovered using 50 μl Dynabeads MyOne Streptavidin C1 beads (Life Technologies, CA, USA). Binding and washing were carried out according to the MYbaits 3.0 manual without the use of Hyb#4. Incubation of captured libraries with streptavidin beads was increased to 45 min. 30 μl molecular biology grade water was used to re-suspend the DNA. The captured libraries were PCR amplified for 18-30 cycles using the KAPA HiFi DNA Polymerase and the protocol for cycling conditions given in the previous paragraph (Kapa Biosystems, MA, USA).

PacBio libraries at MPI were prepared using the ‘2 kb Template Preparation and Sequencing’ protocol (Pacific Biosciences, CA, USA), and size selected for 2-5 kb using a BluePippin instrument (0.75% agarose dye-free/0.75% DF 2-6 kb Marker S1, Start = 2000, End = 2000) (Sage Science, MA, USA). PacBio libraries at UNC were prepared using the manufacturer’s recommended procedure for ‘5 kb Template Preparation and Sequencing’, and size selection for fragments over 3 kb was done using a SAGE-ELF instrument with 0.75% gel cassettes (Sage Science, MA, USA), size-based separation mode, target value 3 kb and target well 10. All wells containing fractions above 3 kb were pooled. PacBio libraries at TSL data were prepared by size selecting fragments > 3 kb from the captured library using a SAGE-ELF instrument as described above.

Quality control of all libraries was performed with Qubit (Life Technologies, CA, USA) and Bioanalyzer (Agilent, CA, USA). The PacBio RS II sequencing platform and P6-C4 chemistry was used to sequence each accession or multiplexed pool on individual SMRT cells (Pacific Biosciences, CA, USA). Sequencing of several accessions was repeated in order to obtain sufficient output (Table S1).

#### *De novo* assembly and NLR annotation

##### Assembly

Reads were assembled with Canu (version 1.3; -pacbio-corrected, trimReadsCoverage = 2, errorRate = 0.01, genomeSize = 2 m; (Koren et al., 2017)). Expected genome size was adjusted to 2 Mb, which reflects the proportion of an *A. thaliana* genome expected to be captured (∼1.4 Mb NLR genes plus expected flanking regions). Read ends were trimmed using a minimum evidence of two reads. Contigs were removed if they were fully contained in a larger contig with > 99.5% identity. For final assembly size and contig length distribution, see https://github.com/weigelworld/pan-nlrome.

##### Annotation

Coding and non-coding elements were annotated. Evidence- and profile-based methods were integrated in the MAKER pipeline (version 2.32; pred_flank = 150, keep_preds = 1, split_hit = 3200, ep_score_limit = 95, en_score_limit = 95; (Campbell et al., 2014)). Genes were predicted with AUGUSTUS (version 3.1.0; defaults; (Stanke et al., 2006)) and SNAP (version 2006-07-28; defaults; (Korf, 2004)). AUGUSTUS used the default ‘*Arabidopsis*’ profile for gene prediction, and SNAP used a custom Hidden Markov Model (hmm) based on NB-ARC and/or TIR containing genes. Gene predictions were improved using Col-0 proteins and transcripts from the Araport11 website (Araport11_genes.20151202.pep.fasta, Araport11_genes.20151202.mRNA.fasta, (https://www.araport.org/)). Protein and transcript evidence was considered only if its mapping quality was sufficiently high (see above for ep_score_limit and en_score_limit). Repeat-masked regions were not used for gene prediction (RepeatMasker; version open-4.0.5; model_org = *Arabidopsis*; (http://www.repeatmasker.org/RMDownload.html)). *Capsella rubella* and *Arabidopsis lyrata* reference annotations were revised to create reliable sets of NLRs for these outgroups.

Reference annotations, evidence and gene predictions were integrated in MAKER. RNA-seq data guided gene prediction with BRAKER1 (version 1.9; defaults; (Hoff et al., 2016)). Reads from silique, root, stem, leaf, and flower (PRJNA336053; PE; 100 bp; 5-10 Mb; (Wang et al., 2016)) were mapped to the reference genomes using HISAT2 (version 2.0.5;–no-mixed–no-discordant; (Kim et al., 2015)). Gene prediction was guided by mapped reads, and these were also used to assemble transcripts (Cufflinks; version 2.2.1; defaults; (Trapnell et al., 2010)). Gene predictions were compared to reference gene annotations using MAKER (pred_gff, model_gff). Evidence mappings were used to choose the best annotation per locus. Reference genomes and annotations were taken from Phytozome (https://phytozome.jgi.doe.gov/). Assembled transcripts acted as the primary evidence (est_gff), re-annotated *A. thaliana* NLR transcripts and proteins were used as alternative evidence (altest, protein).

Protein domains were predicted for gene models and for AUGUSTUS gene-prediction products using Pfam hmms and coiled coils (InterProScan; version 5.20-59.0; -dp -iprlookup -appl Pfam,Coils; (Zdobnov and Apweiler, 2001)). RepeatMasker results were visualized to flag complicated regions. Diverged repeats in outgroups were additionally masked and visualized (repeat_protein = te_proteins.fasta provided by MAKER).

##### Web Apollo

Gene models and evidence tracks from Maker were integrated into WebApollo for manual inspection (version 2.0.4; http://ann-nblrrome.tuebingen.mpg.de; (Lee et al., 2013)). Additional evidence tacks were added to evaluate the quality of the gene models. A track for duplicated and diversified genes was added by aligning transcripts (track = est2genome-50) and proteins (track = protein2genome-50) from the reference gene annotation (Araport11) to each NLRome (–percent 50, exonerate; version 2.2.0; (Slater and Birney, 2005)). The same procedure was carried out on known pseudogene transcripts. Protein domain predictions were added for both MAKER (track = InterProScan) and AUGUSTUS (track = InterProScan Augustus) gene models. A track with CCS read mappings (pbalign; version 3.0; defaults; (Tyagi et al., 2008)) was added to aid contig quality inspection. In case of *A. lyrata* and *C. rubella*, RNA-seq alignment data were added for inspection of intron-exon boundaries.

##### Manual re-annotation

Genes containing NB-ARC or TIR domains were manually inspected (see reannotation SOPs at https://github.com/weigelworld/pan-nlrome). Gene models were evaluated using several biological evidence layers in Web Apollo. Incorrectly fused genes were split, while incorrectly split genes were merged. Col-0 protein and transcript mappings were used to detect wrongly fused gene models or split gene models. Genes were split, if several proteins or transcripts mapped next to each other within one model. Genes were merged, if protein or transcript mappings spanned several models. Both cases were often associated with multiple gene predictions that were inconsistent with each other. Additional features that were used to identify fused genes were extremely long introns, or pseudogene mappings.

Evidence from protein and transcript mappings, as well as RNA-seq read mappings was considered to select the best gene model. Gene structures were corrected, and intron, exon, and UTR boundaries were refined. Alternative splice forms were not used in this study. Genes were flagged with ‘corbound’ if exon-intron structures were changed without direct protein or transcript evidence, and ‘cortrans’ was used, if translation start points were changed (see gff files at https://github.com/weigelworld/pan-nlrome). Exceptions were individually evaluated. Non-canonical splice sites were confirmed using reference proteins and transcripts. Rare erroneous reference annotations were corrected using TAIR10 annotations. Genes were flagged with ‘pseudogene’ if a pseudogene from Araport11 was aligned to the same region. Incomplete genes and uncorrectable annotations were flagged. Genes at contig borders were flagged as ‘truncated’ if confirmed by protein or transcript mappings. Rarely, genes were extensively changed to rescue domain structures. These genes were flagged with ‘mod’. Erroneous gene models due to misassembled contigs were flagged with ‘misassembly’ if base calls were contradicted reliably by CCS read mappings.

##### Mis-annotated genes

A small number of mis-annotated genes, as determined during manual curation, was removed from the final NLRome (see Table S3H).

#### Classification and architectures

We defined as NLR genes those that contained at least an NB, a TIR, or a CC_R_ (RPW8) domain. I.e., LRR or CC motifs alone were not considered sufficient for NLR identification. As a first subdivision (Table S3D), we defined TNLs (at least a TIR domain), CNLs (CC+NB domain), RNLs (at least an RPW8 domain), and NLs (at least an NB domain). The second subdivision defined 25 different groups by the different combinations of TIR, CC, NB, RPW8, LRR, and X (other Integrated Domains [ID]) independent of sequence and number of domains.

As mentioned earlier (Web Apollo section), protein domains were predicted using Pfam HMMs. CC motifs were refined using a majority vote from Coils (2.2.1; InterProScan-defaults; (Lupas et al., 1991)), Paircoil2 (defaults; (McDonnell et al., 2006)), and NLR-parser (v.2; defaults; (Steuernagel et al., 2015)). Coils and Paircoil2 use databases of many known coiled-coils, whereas NLR-parser uses two NLR-specific coiled-coil motifs (motif16 and motif17) (Steuernagel et al., 2015). CC signatures were considered if predictions from at least two methods overlapped. CCs of functional NLRs previously annotated as CNLs were not always confirmed (Table S2G).

An architecture was defined as the collapsed protein domain set, i.e., without making a distinction between genes in which a domain was found once versus multiple times (Tables S2A and S2F). Canonical architectures contain only NB (Pfam accession PF00931), TIR (PF01582), RPW8 (PF05659), LRR (PF00560, PF07725, PF13306, PF13855) domains, or CC motifs (Figure S2). Non-canonical architectures contain at least one ID, as defined in (Baggs et al., 2017). To identify new and recurring domain arrangements, we compared the reference Araport11 Col-0 NLRs with our *A. thaliana* pan-NLRome (without Col-0 RenSeq NLR genes) and with the NLRome of 19 *Brassicaceae* species (Table S2C). Bash and R scripts used to generate Figure 2 are available in CodeOcean (https://doi.org/10.24433/CO.5847249.v1).

##### OG visualization

Unrefined OGs and corresponding metadata were integrated into iTOL (Letunic and Bork, 2016) for visualization and re-inspection (https://itol.embl.de/shared/pan_NLRome). IDs of refined OGs were added to highlight over-clustered OGs and outliers. The domain architecture and the protein length were plotted to compare OG members structurally. Transposable elements (TEs) in exons, introns, and 2 kb up- or downstream of NLRs were integrated into iTOL as well. Sub-clustering might be related to accession-based metadata, thus we included for each protein if its accession belonged to the relict group, the geographic origin, and the admixture group.

##### *Albugo candida* screening

A suspension of 10^5^ *Ac*Ex1 zoosporangia/mL in distilled water was sprayed on four 4-week old plants of each accession (*Ac*Ex1: (Prince et al., 2017)). Phenotypes (green resistant: GR, green susceptible: GS, weak chlorotic susceptible: WCS) were recorded at 10 days post inoculation, with two replicate experiments. Several accessions exhibited delayed/poor germination and were not tested (N/A). Results can be found in Table S1.

##### Identification of paired NLRs and sensor-executor pairs

We generated a list of paired NLR genes containing the nine Col-0 divergently transcribed TNLs sharing a genetic arrangement similar to the *RPS4*/*RRS1* pair (Narusaka et al., 2009). We added seven additional divergently transcribed pairs identified by manual inspection of 138 Col-0 genes that contained a TIR domain. We also used an in-house CNL list to mine the Col-0 genome for consecutive genes and included six paired CNL-CNL loci, of which only two are divergently transcribed. During manual curation, we further identified one divergently transcribed pair of TNLs with no Col-0 allele.

To further examine pair evolution, we narrowed the list of pairs to ones in head-to-head orientation in either the Col-0 reference genome, or in the RenSeq assemblies, and to those that were phylogenetically placed in the clades with the RPS4 or SOC3 executor TNLs or in the clades with the RRS1 or CHS1 sensor TN(L)s. The NB domain alignment-based phylogeny used for this decision is shown in Figure S6. Of 16 such pairs in the pan-NLRome, two are missing from the Col-0 reference genome.

To test the possibility that genetic proximity could lead to co-evolution or conservation of population genetic characteristics, we identified a set of control NLR pairs that are less than 4 kb apart in the Col-0 reference genome. We identified 15 such pairs; for a list of all pairs specific to this context and pertaining to Figure 7B, see Table S4B.

##### Figure generation

Bash and R scripts used to generate Figure 2 and Figure 4 are available in CodeOcean (Monteiro, 2019) (https://doi.org/10.24433/CO.5847249.v1). All quantitative data panels were generated using R (version 3.4.4; (R Development Core Team, 2008)) and RStudio (RStudio Team, 2015), unless otherwise stated. For clarity, floating text was added to SVG files generated in R using Inkscape (version 0.92.3; https://inkscape.org). Used packages included ggplot2, grid, gridExtra, reshape2, gsubfn, cowplot, rworldmap, yarrr, UpSetR, PerformanceAnalytics, karyoploteR and viridis (see Key Resources Table and our Github repository). OG phylogenetic trees were visualized using iTOL (Letunic and Bork, 2016). The phylogeny for Figure S6 was generated through the use of MEGA 6.06 including MUSCLE (Edgar, 2004) and WAG maximum-likelihood phylogenetics (Whelan and Goldman, 2001). Figures 7C and S6 were generated using FigTree (v1.4.3; https://github.com/rambaut/figtree). Input data and R scripts for all relevant figures can be found at https://github.com/weigelworld/pan-nlrome. Adobe Illustrator CS6 v16.0.4 was used at the final stage to edit and compose main and supplemental figures.

#### Quantification and statistical analysis

##### Read correction

We used PacBio raw reads that cover the same genomic DNA fragment multiple times (circular consensus sequencing). The raw reads were self-corrected to consensus reads, which reduces the read error from 17% to 2% (CCS; version 2.0.0; default settings; (Travers et al., 2010)). Where indexing was employed, corrected sequences were de-multiplexed (see demultiplexing script at https://github.com/weigelworld/pan-nlrome/). A single, combined CCS dataset was created for accessions that were sequenced on more than one SMRT cell. Only CCS reads with > 99% per-base accuracy were considered (see https://github.com/weigelworld/pan-nlrome for final read statistics).

#### Assembly Validation

##### Quality Scores

We mapped CCS reads back to the assembly. Any read that is not mapped to its correct origin because the NLR was not assembled, is expected to map to a sequence related NLR gene (if such a gene is present), giving rise to pseudo-heterozygous SNP calls. For read mapping, a pseudo-genome was constructed for each RenSeq assembly by combining the assembled contigs with NLR gene-masked chromosome sequences from the TAIR10 reference genome. On the RenSeq contigs, non-NLR genes were masked, as almost all should be present in the reference genome. CCS reads were then mapped to these pseudo-genomes (minimap2; 2.9-r748-dirty; -x map-pb (Li, 2018)). SNPs were called for NLR genes using high quality mappings only (htsbox pileup; r345; -S250 -q20 -Q3 -s5; available at https://github.com/lh3/htsbox).

To assess assembly quality, the number of pseudo-heterozygous sites (hetsites) was compared to the total number of mappable NLR gene bases (totalsites). The quality was calculated as logarithmically linked to the ratio of pseudo-heterozygous calls to the total amount of mapped bases.

$$Q=abs\left(-10\text{∗}log10\left(\frac{hetsites}{totalsites}\right)\right)$$

##### Completeness assessment

We used assemblies of subsampled reads from the Col-0 reference accession, for which the ground truth is known, to assess assembly completeness. Corrected CCS reads from Col-0 were sub-sampled from 100 to 1% in 1% steps (seqtk sample; v.1.0-r82-dirty; defaults). 100% of the data correspond to 26,639 reads with a N50 read length of 2,846 bases and 77.98 Mb total sequence. Each sub-sampled dataset was assembled with Canu as described above. All genes from the original 100% RenSeq Col-0 assembly were mapped to each sub-assembly to detect assembled NLRs. NLR genes were extracted from these alignments (exonerate; v.2.2.0;–model est2genome–bestn 1–refine region–maxintron 546; (Slater and Birney, 2005)). The quality of each sub-assembly was assessed based on pseudo-heterozygous calls as described above.

For each sub-assembly, we determined what fraction of the full Col-0 reference NLR complement had been recovered. NLR gene models were evaluated using rnaQUAST (version 1.5.0; defaults; (Bushmanova et al., 2016)) with the TAIR10 reference genome and NLR genes annotated in Araport11. Completeness was calculated by dividing the amount of covered NLR genes (in bases) by the total length of the Araport11 NLR genes. The relation between completeness and quality (as defined in the section above) of the tested Col-0 sub-assemblies was used to infer completeness values for the other accessions. Each accession quality was used to find the corresponding completeness value from the tested Col-0 sub-assemblies.

##### Similarity to Col-0 reference accession

We determined if the similarity of an accession to the Col-0 reference accession influenced its quality. RenSeq assemblies were mapped against the Col-0 assembly (minimap2; 2.9-r748-dirty; defaults; (Li, 2018)) and SNPs were called in NLR gene regions (htsbox pileup; r345; defaults; available at https://github.com/lh3/htsbox). Only biallelic SNPs were used to calculate the Identity-By-State (IBS) value for each accession compared to Col-0 (SNPRelate_1.10.2; method = ’biallelic’; (Zheng et al., 2012)). For assembly validation results, see https://github.com/weigelworld/pan-nlrome.

#### Pan-NLRome generation

Our *A. thaliana* pan-NLRome was constructed using a protein-clustering approach, resulting in ‘orthogroups’ (OGs) (Table S3E). Clusters were generated with a three-step procedure. First, all-against-all full length protein alignments were produced (DIAMOND; version 0.9.1.102;–max-target-seqs 13169–more-sensitive–comp-based-stats; (Buchfink et al., 2015)). Second, putative ortholog and inparalog relationships were identified (orthAgogue, commit 82dcb7aeb67c,–use_scores–strict_coorthologs; (Ekseth et al., 2014)). Third, protein clusters were formed based on the orthology information (mcl; version 12-135; -I 1.5; (Enright et al., 2002)). OGs had to contain at least two genes; the rest were considered as singletons.

##### Orthogroup (OG) refinement

The initial set of OGs was inspected for over-clustering by screening for paralogs within OGs. Protein alignments were generated for each OG with > 4 members (T-Coffee; version 11.00.8cbe486; mode: mcoffee; (Neelabh et al., 2016)) and converted into the corresponding codon alignments (PAL2NAL; version 14, defaults; (Notredame et al., 2000)), which were used to remove three different types of outliers: non-homologous, partly mistranslated and low similarity sequences (OD-seq; version 1.0;–analysis bootstrap; (Jehl et al., 2015)). The remaining core sequences for each OG were realigned in protein space, converted into the corresponding codon alignments and used to infer a phylogenetic tree (FastME; version 2.1.5.1; -s -n -b 100; (Lefort et al., 2015)). Each tree was used to detect simple paralogs (duplications in terminal branches) and complex paralogs (duplications spread across the whole phylogeny). For OGs where at most 5% of the accessions with at least one OG member showed evidence of within-OG duplications, all paralogs were removed. Otherwise the tree was split at (accession) duplication events (ete3; version 3.0.0b36; (Huerta-Cepas et al., 2016)), and new OGs were created from the leaves of all resulting sub trees (Table S3F). Codon alignments and trees were re-computed using ExaBayes version 1.4.1 with default settings (Aberer et al., 2014) and considered robust with all sampled parameters showing an effective sample size (ESS) over 200. Final consensus trees were generated with a burn-in of 25% and MRE as thresholding function (see alignments and trees at https://github.com/weigelworld/pan-nlrome).

##### Saturation analysis

OG as well as haplotype and nucleotide diversity discovery rates were determined by saturation analysis. For OG discovery, accessions were randomly selected from the pan-NLRome and the number of OGs counted they were part of. The process was repeated 1,000 times starting with two and ending with 64 randomly selected accessions. For nucleotide and haplotype diversity discovery, accessions were selected as above. Nucleotide and haplotype diversity were calculated for each of the replicates and averaged. The process was repeated 100 times starting with two and ending with 64 randomly selected accessions.

##### OG classification

The final set of refined OGs was annotated with metadata derived from transcript-based majority votes (e.g., classes), transcript-based counts (e.g., members with IDs, members flagged as paired, members flagged as clustered) or OG-based counts and analysis (e.g., type, diversity statistics, positive selection, average tree branch length). Refined OGs were classified into three size-based categories after visual inspection of OG size density distribution (Figure 3A): < 13 members as “cloud,” > 51 members as “core,” and 13 **≤** OG members **≤** 51 as “shell.” OGs were classified as clustered if the majority of OG members were annotated as clustered. OGs were classified as ID-containing if at least one member contained an ID. We further classified OGs using protein domain architectures using the majority vote from domain architectures of OG members.

#### Diversity, selection, association and expression analyses

Diversity and neutrality statistics were calculated for each codon alignment of the refined OGs (PopGenome; version 2.2.4; (Pfeifer et al., 2014)). Domain-specific diversity statistics were calculated on subset, concatenated alignments only consisting of positions covering the respective domains (e.g., NB). Alignment columns were annotated with a majority vote across all individual sequence annotations and selected subsequently. The average tree-derived branch length for an OG was defined as the sum of all branch lengths normalized by the OG size. Positive selection tests were carried out using HyPhy (version 2.3.13; (Pond et al., 2005)) using codon alignments and corresponding trees. Pervasive diversifying positive selection was detected with FUBAR (version 2.1; default parameters, (Murrell et al., 2013)) and sites considered with a posterior probability > = 0.95 (Tables S4C and S4D). Episodic diversifying positive selection was detected with MEME (version 2.0.1; default parameters; (Murrell et al., 2012)) and sites considered with a p value threshold ≤ 0.01 (Tables S4E and S4F). Branch-site selection was detected with aBSREL (version 2.0; (Smith et al., 2015)) and branches considered under selection at a p value of 0.01 having at least five members. Invariable codons were identified using a custom script (see *msa2cns* script at https://github.com/weigelworld/pan-nlrome). Domain-specific positive selection was calculated on a subset of positions covering the respective domains (e.g., NB). The alignment annotation was the same as for the Domain-specific diversity statistics.

Statistical comparison of OGs grouped by established functional role (Figure 7A) was performed by one-way ANOVA and post hoc Tukey test using the online astatsa.com resource. One-way ANOVA performed on four groups (biotroph:adataped, biotroph:non-adapted, hemibiotroph and helper) of combined size 30 within-OG nucleotide diversity measurements yielded a p value of 1.1172e-05. The post hoc Tukey test revealed that the biotroph:adataped group was significantly different to each other group individually with p values < 0.01. All the other groups were statistically indistinguishable from each other.

Assessment of the relationship of pair status and nucleotide selection (Tajima’s D) (Figure 7B) was performed using linear regression in Excel for Mac v15.33 (Microsoft Corporation, MA, USA). 15 control pairs and 16 sensor-executor pairs of OGs were identified and tested for correlation. The list of pairs and full analysis is presented in Table S4B.

An average expression percentage was estimated for each OG using RNA-seq data from the 1001 Genomes Project (1001 Genomes Consortium, 2016, Kawakatsu et al., 2016). For each accession, a pseudo-transcriptome was generated from accession-specific NLR transcripts plus all non-NLR transcripts from the Col-0 reference accession. NLR gene introns were added to the pseudo-transcriptome for expression filtering. Transcript abundance was quantified with pseudo-alignments of RNA-seq reads from 727 accessions (kallisto, v.0.43.0,–single -l 200 -s 25 -b 100–bias; (Bray et al., 2016)). The data was further processed with R (v.3.4.1). Abundance was normalized (DESeq2; v.1.16.1; estimateSizeFactor; (Love et al., 2014)) and expressed NLR genes were defined using a per-accession expression threshold. Expression counts from introns were used to compute a background expression density distribution and subtracted from the density distribution of all NLR expression counts. The lowest expression level with a density > 0 was used as minimum expression threshold. On average, NLRs were considered expressed with an expected count ≥ 175. Finally, for each NLR, the percentage of accessions that provided reliable expression was calculated.

We consulted the AtGenExpress expression atlas to gauge absolute expression level (Schmid et al., 2005), bias in leaf versus root specificity of expression and the pathogen inducibility of Col-0 NLRs. NLR genes were broadly divided into low-, medium- and high-expression groups, based on whether at least two samples had absolute signal values in the developmental datasets that were 20 < expression < 100, 100 < expression < 1,000, 1,000 < expression. Genes that had generally absolute signal values below 20 were characterized as marginally expressed. If average expression in leaf and rosette samples was at least twice of that in root samples, or vice versa, genes were considered tissue biased in expression. Note that differences between tissues can be much larger, > 100 fold. Pathogen inducibility was assessed from the AtGenExpress pathogen dataset (Kemmerling et al., 2011), based on induction by at least two pathogen-related stimuli. The final Col-0 NLR annotation was amended for the respective OGs.

OG topologies were tested for association with the above mentioned metadata using BaTS (version 0.10.1; 100 bootstraps, burn-in 25%). Associations were considered with a BaTS significant value ≤ 0.01. Associations results were overlaid with branches under episodic diversifying selection by comparing BaTS p values of selected versus unselected branches respectively its members. BaTS significant values were mapped to members using the metadata supplied to BaTS. Both p value sets were compared using a Wilcoxon rank sum test and considered significant at a p value ≤ 0.01 and the selected branch having smaller significant values than the unselected one (https://github.com/weigelworld/pan-nlrome/blob/master/code/possel2bats.py).

We assessed the probability of non-clonal evolution (i.e., intragenic recombination or gene conversion) in OGs by running 3seq build 170612 (Lam et al., 2018) in full mode, inspecting only distinct sequences on OG nucleotide alignment multifasta files. OGs with a corrected p value of < 0.05 were considered to have evidence of recombination/gene conversion events.

##### Placement of non-reference OGs

Annotated non-NLR proteins in the 64 accessions were clustered into OGs using the approach described above (see ‘pan-NLRome generation’ section). Briefly, non-NLR protein clusters were generated with three main procedures. First, we used DIAMOND to obtain all-against-all full length protein alignments (DIAMOND; version 0.9.1.102;–max-target-seqs 50691–more-sensitive–comp-based-stats; (Buchfink et al., 2015). Second, we identified putative orthologs using orthAgogue (orthAgogue, commit 82dcb7aeb67c,–use_scores–strict_coorthologs (Ekseth et al., 2014). Third, we used the MCL algorithm to define the cluster structure of the similarity relationships established in the previous steps (mcl; version 12-135; -I 1.5 (Enright et al., 2002). No refinement steps were applied to non-NLR OGs (Table S3I).

For each NLR gene in NLR-OGs, we tested contig linkage with other annotated genes in the respective accession. To establish OG-OG co-occurrence, we extracted OG size (node size), NLR-OGs and non-NLR-OGs (node color). Whenever OGs contained a Col-0 allele we established a reference anchoring position in the reference genome (node shape).

OG co-occurrence matrices were used to calculate bidirectional networks of contig linkage at different thresholds ( **≥** 10 shown in Figure S3) using Cytoscape v.3.5.1 (Shannon et al., 2003), running on Java v. 1.8.0_151. We extracted all observed combinations from the accession’s gff files and visualized co-occurrence intersections in UpSet plots. Apparent paired NLR genes were identified from annotation flags (see ‘Identification of paired NLRs and sensor-executor pairs’ and ‘Analyses of over-represented flags’ sections; Table S3G). All enrichments with a q-value below 0.1 (Fisher’s Exact and hypergeometric tests/FDR) are reported. Positions of anchored OGs are shown as a schematic karyogram (see ‘Figure generation’ section). Col-0 reference NLR gene coordinates were extracted from TAIR9/Araport11 annotation. Non-reference OG anchoring positions are approximate values derived from manual inspection of NLRome assemblies (see ‘Web Apollo’ section). Bash-, R-scripts and input files for the UpSet plots, karyogram and the Cytoscape network are available in CodeOcean (https://doi.org/10.24433/CO.5847249.v1).

##### Analyses of over-represented flags

Gene annotation flags (such as ‘paired’, ‘fusion’, ‘merged’) in each OG were compiled using an in-house bash script. Flag enrichment was calculated in R using hypergeometric test. Multiple testing was corrected via false discovery rate (FDR) estimation and q-values below 0.1 were reported (Table S3G).

### Data and code availability

The data generated during this study are available at the European Nucleotide Archive (ENA): PRJEB23122. The code generated during this study are available along with manually curated gene models (gff), domain annotations, OGs, protein and transcript alignments, phylogenetic trees, scripts necessary to produce figures and further metadata files containing information parsed and restructured from the supplemental tables in this manuscript at the GitHub pan-NLRome repository (https://github.com/weigelworld/pan-nlrome/). Assemblies are available for download via the 2blades foundation (http://2blades.org/resources/). Visualization of OG phylogenetic trees and metadata is available at iTOL (https://itol.embl.de/shared/pan_NLRome).

### Additional Resources

A genome browser is available at http://ann-nblrrome.tuebingen.mpg.de.

## Web Resources

Genome browser, http://ann-nblrrome.tuebingen.mpg.de

## Supplemental Information

Table S1. Accession Metadata, Related to STAR Methods Metadata for 64 *Arabidopsis thaliana* accessions.

Table S2. Architectures, Related to Figure 2 (A) Domains in the *A. thaliana* Pan-NLRome. (B) Universe of IDs detected in the *A. thaliana* Col-0 reference accession and/or reference NLRomes of 19 other Brassicaceae species. (C) Sources of the 22 proteomes from 19 Brassicaceae species used to generate the Brassicaceae NLRome. (D) Domain architecture prevalence, number of paired NLRs and presence in the *A. thaliana* Col-0 reference accession and/or 19 Brassicaceae. (E) Number of architectures detected for each of the 27 IDs already known from Brassicaceae NLRomes. (F) Architecture metadata. (G) Prediction of CC motifs in functionally validated CNLs from Col-0 reference accession.

Table S3. Annotation, Related to Figures 1–4 (A) Gene models included in the *A. thaliana* pan-NLRome. (B) Gene models used to generate the Brassicaceae NLRome. (C) Number of non-reference OGs that can be anchored to Col-0 genomic positions through co-occurrence with reference OGs on the same contigs. (D) First and second subdivisions used to define NLR classes. (E) Homology-based clustering into OGs without refinement. (F) Refined final set of OGs. (G) Enrichment of curation flags in OGs (only q-values <0.1). (H) Mis-annotated genes manually removed from the NLRome. (I) Non-NLR OGs used for non-reference OG anchoring.

Table S4. Selection Analyses, Related to Figures 5–7 (A) Frequency of positively selected sites in IDs. (B) List of OGs categorized as sensor/executor or control pairs, with functional metadata and Tajima’s D statistics. (C) Results from pervasive diversifying positive selection scans. (D) Posterior probabilities of diversifying positive selection by position. (E) Results from episodic diversifying positive selection scans. (F) P values for episodic diversifying positive selection by position. (G) Results from the 3seq analysis to assess the probability of non-clonal evolution in each OG. (H) Association of specific branches within OGs with metadata. (I) Association of overall OG phylogenetic topology with metadata.

Table S5. Oligonucleotides, Related to STAR Methods (A) Oligonucleotides for custom barcoded adapters in TSL accessions. (B) Bait library (v2.4).
